# Off-target drug repurposing for rare and ultra-rare cancers: towards a scalable therapeutic framework

**DOI:** 10.3389/fgene.2026.1921857

**Published:** 2026-09-17

**Authors:** Gehan Premaratne, Malaka Ameratunga

**Affiliations:** 1 Alfred Hospital, Melbourne, VIC, Australia; 2 Department of Cancer Medicine, Monash University, Melbourne, VIC, Australia

**Keywords:** drug development, off-target, repurposing, biomarker, precision oncology, rare cancers, next-generation sequencing

## Abstract

Drug repurposing represents a pragmatic strategy for patients with cancer who have limited standard-of-care options. Traditional *in silico* or functional repurposing approaches often rely on well-characterised protein targets or validated experimental models, which are sparsely available in rare cancer settings and impractical within clinically relevant timeframes. Tumour-agnostic biomarker-driven strategies, by contrast, offer more timely workflows for integration into routine oncology practice, but have largely been dominated by ‘on-target’ approaches that may be less applicable to rare cancers frequently lacking canonical targetable mutations. However, emerging pharmacological evidence demonstrates that many anticancer agents engage a broader target landscape, creating opportunities for ‘off-target’ repurposing against non-canonical tumour dependencies. Although less established, we contend ‘off-target’ repurposing remains a potentially important strategy offering therapeutic hope to patients with rare cancers for whom no standard options, targetable biomarkers, or disease-specific trials exist. This perspective explores emerging evidence surrounding ‘off-target’ repurposing as a strategy, as well as evaluating recent criticisms of off-target low-evidence genomic matching in precision oncology literature. By doing so, we advocate for development of rigorously designed prospective clinical trials to examine ‘off target’ strategies, centred in safety of participants, evidence generation, and accessibility. Such frameworks require embedding off-target candidates within trial designs suited to small heterogeneous cohorts, with built-in mechanisms for evidence generation and access to therapies. We propose safe implementation should incorporate longitudinal monitoring, and parallel orthogonal validation to strengthen biological and pharmacological plausibility, refine drug and dose selection, and inform adaptive treatment modification over time.

## Introduction

Rare and ultra-rare cancers, malignancies with an annual incidence below 6 per 100,000 and 1 per 1,000,000 respectively, account for a substantial proportion of cancer diagnoses and mortality, yet remain disproportionately underserved by research and drug development efforts (Stacchiotti et al., 2021; De Heus et al., 2022). Most rare and ultra-rare cancers cannot be studied within the context of randomised trials due to shortage of cases and high biological heterogeneity, only amenable to limited prospective cohort studies, and small single-arm studies (Pushpakom et al., 2019). Additionally pharmaceutical investment in drug development for rare and ultra-rare cancers is usually low, given the resource cost of adequately powered trials, geographically isolated patient population, and limited return on investment (Alvi et al., 2017).

Drug repurposing is the systematic evaluation of existing FDA-approved drugs for off-label anti-cancer activity; an attractive option in rare and ultra-rare cancer contexts due to established safety profile, pharmacokinetic profiling, and reduced cost-to-clinic time (Stacchiotti et al., 2024). Most existing drug repurposing frameworks depend on ‘bottom-up’ in-silico matching of drug candidates to drug targets, or functional ‘top-down’ experimental screening of drug candidates (Recino et al., 2025). Whilst promising, these exhaustive drug repurposing platforms have not achieved widespread adoption by the FDA. From a practical standpoint, many existing drug repurposing pipelines are too slow to reliably inform clinical decision-making, often extending on the order of over several weeks-to-months, which may miss critical therapeutic decision windows and delay treatment permitting disease progression. Additionally, these approaches often assume mature protein characterisation of the tumours, or validated experimental tissue models which are sparsely available in rare and ultra-rare tumour settings (Subbiah et al., 2025).

Recent advances in precision oncology have enabled tumour-agnostic repurposing methods, which rely on biomarkers and begin with genomic data already generated in routine clinical care (e.g., comprehensive gene panels). These broadly comprise ‘on-target’ repurposing in which established biomarker-drug relationships are extended across tumour types, and the more recent ‘off-target’ repurposing approach which seeks to identify therapeutic vulnerabilities beyond established biomarkers (Walpole et al., 2024). Although both strategies have promise, their efficacy remains context-dependent. This perspective seeks to develop a stratified framework for drug repurposing particularly as it pertains to rare and ultra rare cancers. For such individuals with limited or no therapeutic options remaining, we position ‘off-target’ repurposing as a rational path forwards; with expanding opportunities as next-generation sequencing (NGS/WES) is increasingly adopted for patient care. Secondly, we contextualise recent criticisms of ‘off-target’ matching approaches in the literature, highlighting the methodological limitations of existing retrospective analyses. In doing so, this perspective advocates for the systematic development of prospective clinical trials evaluating the potential of ‘off-target’ repurposing. Follow-on recommendations are provided for clinical trial structures that enable such low-evidence off-target therapeutic hypotheses to be safely trialled in participants, prioritising pathways for systematic evidence generation and equitable access.

## Limitations of traditional drug repurposing for rare cancers

Traditional drug repurposing approaches include *in silico* drug screening or experimental functional screening approaches. These methods assume biologically well-characterised tumours or availability of validated experimental tissue models, both of which are uncommon in rare and ultra-rare cancer settings and often untenable within clinically relevant timeframes (Xia et al., 2024). In-silico drug screening encompasses a broad spectrum of computational strategies that interrogate tumour protein profiles against libraries of approved drugs to identify repurposing candidates (Park, 2019). This includes molecular docking, network-based modelling, and expression-signature analyses (Cavalcante et al., 2024; Tanoli et al., 2025). However, the core paradigm of pairing one or more drug(s) with a target for each evaluation creates substantial scalability constraints as drug libraries expand and more disease-targets are discovered. This combinatorial complexity limits the utility of these approaches in oncology workflows, where timely therapeutic decisions must be made.

Similarly experimental phenotypic and functional screening approaches, which rely on empirical testing of drug libraries in tumour-derived cellular or tissue models, face major limitations in rare and ultra-rare cancers (Letai et al., 2022). These malignancies frequently lack robust *in vitro* systems such as established cell lines or patient-derived organoids (PDOs), reflecting both the paucity of dedicated research and limited availability of biobanked tissue samples (Van Renterghem et al., 2023; Letai, 2017; Corsello et al., 2020). Nonetheless, several model-independent functional approaches have shown early promise in this setting. These include *ex vivo* phenotypic screening platforms which utilise a direct patient biopsy for drug screening (Phan et al., 2019; Williams et al., 2022), as well as genetic perturbation strategies such as CRISPR and RNA interference knockdown screens to prioritise drug candidates for cancer therapeutics (Behan et al., 2019; Tsherniak et al., 2017). However, despite advances in multiplexed and high-throughput laboratory platforms, experimental screening approaches remain intrinsically resource-intensive, time-consuming and limited by hardware capability.

## Biomarker-based repurposing: “on-target” approaches and pitfalls

Tumour-agnostic repurposing pipelines, rooted in knowledge of biomarkers (i.e., canonical tumour drivers that are targetable by existing drugs), represent an alternative to traditional drug repurposing paradigms. The most well-known approach is ‘on-target’ repurposing, whereby a drug originally developed for one cancer type is extended to treat another tumour containing the same genetic alteration, identified through the patient’s routine comprehensive genomic panel (CGP) or broader next-generation sequencing techniques (WES/WGS). This approach of using a drug to target the same biological pathway, but across tumour types, is well established within precision oncology. For example, ALK inhibitors originally used for ALK positive lung cancer, have been extended to treat neuroblastoma and large cell-lymphomas containing ALK-rearrangements (Gambacorti-Passerini et al., 2011; Mossé et al., 2013). Similarly, BRAF inhibitors initially developed for melanoma have demonstrated efficacy across multiple BRAF-mutant malignancies, resulting in tumour-agnostic FDA approval except in colorectal cancer (Vranic et al., 2023).

While ‘on-target’ repurposing has achieved important clinical successes in precision oncology, accumulating evidence suggests that its efficacy is heterogeneous between tumour types and is not truly tumour-agnostic. The DRUP (Drug Rediscovery Protocol) trial provides a large-scale, prospective evaluation of this phenomenon (Verkerk et al., 2026). Post-hoc analysis of outcomes revealed tumour type significantly influenced clinical benefit rate in 4 out of 17 biomarker-drug subgroups. For example, MET-amplified cancers treated with crizotinib demonstrated superior outcomes in lung cancer compared with tumours arising in other tissues, and PARP-inhibitors showed greatest benefit in prostate cancer compared to other malignancies (Verkerk et al., 2026). This tumour-specific heterogeneity of ‘on-target’ biomarker-matched therapy can be understood by considering that treatment response is modulated not only by the presence of a targetable mutation, but also by broader biological context including signalling pathway redundancy, co-occurring genomic alterations, and tumour microenvironmental factors (Passaro et al., 2024). This is exemplified by BRAF V600E which is highly targetable in melanoma, yet single-agent BRAF inhibition performs poorly in colorectal cancer because of paradoxical EGFR signalling re-activation (Prahallad et al., 2012). Hence shared mutation does not always imply shared therapeutic vulnerability, instead biomarker ‘on-target’ matching must be understood as tumour and context-sensitive.

## “Off-target” repurposing in rare cancers

However, an important question remains: what approach should be taken when patients have limited standard of care options available or lack canonical targetable mutations - as is frequently encountered in rare and ultra-rare cancers? For this population, we propose ‘off-target’ repurposing as an alternative tumour-agnostic approach that may offer pragmatic therapeutic opportunities where none otherwise exist (Palve et al., 2021). Off-target repurposing aims to identify existing drugs with potential activity against non-canonical dependencies. Its biological premise is supported by growing evidence that many targeted anticancer agents interact with a substantially broader range of proteins than their canonical target. Large-scale experimental profiling has demonstrated extensive and previously unrecognised off-target interactions among clinically-used anti-cancer agents, including kinase inhibitors and PARP inhibitors (Antolin et al., 2020; Klaeger et al., 2017). This ‘off-target’ framework substantially broadens the repurposing search space with novel potential drug interactions that may otherwise remain unexplored, and may translate into clinically meaningful therapeutic opportunities (Palve et al., 2021). For example, Crizotinib, initially developed as a MET inhibitor, was subsequently recognised to also inhibit ALK and ROS1, with exploitation of these additional targets yielding clinical benefit in ALK- and ROS1- rearranged non-small cell lung cancer (Shaw et al., 2013; Shaw et al., 2014).

Building on this experimental foundation, Walpole et al. (2024) introduced a scalable framework for identifying off-target repurposing opportunities in patients with advanced cancers and limited therapeutic options (Walpole et al., 2024). Beginning with comprehensive genomic profiling (CGP), likely pathogenicity and gain-of-function (GOF) mutations are prioritised before excluding canonical biomarkers for which established targeted therapies already exist (e.g., EGFR, BRAF, ALK, HER2) (Antolin et al., 2018; Lin et al., 2021; U.S. Food and Drug Administration, 2026; Shihab et al., 2013). The remaining variants are then screened against pharmacological databases of drug-target interactions to identify FDA-approved drugs capable of engaging these less conventional but potentially actionable genomic dependencies, with further refinement with orthogonal functional/experimental evidence. Using this approach, Walpole et al. identified repurposing opportunities in approximately 14% of patients with otherwise limited treatment options, while maintaining scalability relative to traditional exhaustive screening approaches (Walpole et al., 2024). As new drug-target interactions are discovered in literature, off-target strategies benefit as removal of druggable mutations systematically refines the repurposing search space, thereby increasing sensitivity to detect off-target repurposing opportunities. Broader adoption of next-generation sequencing (WES/WGS) may similarly expand the pool of non-canonical dependencies available for off-target repurposing (Van Pu et al., 2026).

Although ‘off-target’ repurposing offers a promising framework for identifying novel therapeutic opportunities, recent studies have questioned the clinical utility of low-evidence genomic matching. Lin et al. (2026) stratified biomarker-drug pairs according to level of evidence in patients with advanced rare cancers and showed that only biomarker-matched therapies supported by tier 1–3A (high-to-moderate) evidence led to survival benefit, with no overall benefit for low tiers of evidence (3B-4) (as the case for all ‘off-target’ recommendations) (Lin et al., 2026). These findings appropriately argue against substituting weakly supported matched therapies for established treatment, but should also be interpreted within the clinical context in which they were generated. Lin et al. (2026) compared genomically matched therapy to standard-of-care non-matched treatments and disease-specific clinical trials, a comparison that may be appropriate for common malignancies. However, these conclusions may be less applicable to rare and ultra-rare cancers, where standard-of-care options are frequently absent or exhausted early in disease course, with limited access to disease-specific clinical trials. Furthermore, many rare and ultra-rare cancers do not harbour canonical actionable biomarkers (Lucchesi et al., 2018). In this context, off-target repurposing should not be viewed as competing with evidence-based care, but rather as offering a biologically rational and meaningful therapeutic opportunity, in the absence of any viable alternative.

Interpretation is further complicated by therapeutic heterogeneity and the retrospective nature of most studies including ‘off-target’ genomic matching. Without prospective stratification by tumour type and drug class, it is difficult to disentangle whether observed outcomes reflect the true potential of off-target repurposing strategies or the underlying tumour biology in which they were evaluated. For example, many lower-tier (Tier 3B-4) recommendations examined by Lin et al. include drug classes whose efficacy is recognised to be highly tumour-specific such as CDK4/6 inhibitors, PARP inhibitors, immune checkpoint inhibitors, and mTOR inhibitors (Lin et al., 2026). This means outcomes may reflect limitations of specific drug classes within tumour-contexts rather than efficacy of off-target repurposing as a whole.

A related consideration is whether off-target interactions, which may occur at lower binding affinity than the canonical target, necessitate higher drug exposures or dose escalation. However, lower off-target affinity does not automatically imply that a higher clinical dose is required; rather, the relevant question is whether the concentration required for meaningful cellular off-target engagement is achievable at established clinical dosing. Antolin et al. (2020) provides an example of pharmacologically plausible off-target activity at established clinical dosing (Antolin et al., 2020). For comparison, canonical PARP inhibition by rucaparib and niraparib usually occurs at EC50 of low-nanomolar concentrations (Antolin et al., 2020). Antolin et al. showed that rucaparib and niraparib inhibit several off-target kinases at cell target-engagement EC50 values of 223 nM for rucaparib-CDK16 and 209 nM for niraparib-DYRK1A (Antolin et al., 2020). However at their approved clinical doses, steady-state plasma Cmax values for rucaparib and niraparib were approximately 2–9 μM and 3–4 μM respectively (Antolin et al., 2020). Thus, although these off-target interactions were less potent pharmacologically than canonical PARP inhibition, clinically used doses were already well above the concentration needed to engage their intracellular targets in the experimental cell-assay; dose escalation would therefore not necessarily be required simply because the interaction was non-canonical.

Taken together, the conclusions drawn from such studies should not be interpreted as a blanket argument against ‘off-target’ repurposing. For patients with rare and ultra-rare cancers who lack both standard therapeutic options and canonical actionable biomarkers, off-target repurposing remains a logical strategy that requires development of structured frameworks for prospective evaluation.

## Trial structures for safe “off-target” repurposing in rare cancers

Although not specifically designed to evaluate ‘off-target’ repurposing, I-PREDICT and DRUP trials provide compelling examples of how low-evidence genomically guided off-label therapies can be safely implemented within a clinical trial setting (Verkerk et al., 2026; Sicklick et al., 2019; Sicklick et al., 2026). In I-PREDICT, genomic profiling was used to guide personalised multi-drug repurposing regimens, including previously unstudied combinations without established safety or dosing data (i.e., lower tier evidence). Through adaptive dosing and clinical outcome tracking in an N-of-1 trial design, the study demonstrated acceptable safety profiles (Sicklick et al., 2026). DRUP further establishes this through a prospective basket trial structure, providing controlled access to off-label repurposing candidates in patients with advanced cancers lacking standard options, and systematically tracking efficacy across molecularly defined cohorts for evidence generation (Verkerk et al., 2026). Building on these models and others, several key principles emerge for designing clinically implementable ‘off-target’ repurposing trials (Figure 1):

- **Strict patient selection:** Eligible patients should have exhausted or lack standard-of-care options, lack canonical targetable mutations, and not have access to disease-specific clinical trials - as is often the case for rare and ultra rare cancers. This is crucial for identifying patients for whom low-evidence off-target drug candidates may offer clinical value. Importantly, this framework explicitly excludes patients with effective non-targeted standard therapies available, in line with evidence from Lin et al. (2026) demonstrating that low-tier biomarker-drug matches should not be considered when robust standard-of-care treatments exist (Lin et al., 2026).
- **Prospective trial designs suited for rare and ultra-rare cancers:** Embedding ‘off-target’ repurposing within prospective trials serves two main purposes. Firstly prospective design permits in-built stratification within the trial structure according to drug class, and tumour type and biology, facilitating clearer interpretation of treatment efficacy and helping to distinguish the true potential of off-target repurposing from underlying biological heterogeneity (i.e., drug class specificity, signalling redundancy, and tumour microenvironmental influences). Secondly, prospective trials using evidentiary frameworks better suited to rare cancer contexts such as N-of-1, basket, and Bayesian adaptive trials allow for structured capturing of outcome data for repurposed off-label therapies (Subbiah et al., 2025; Verkerk et al., 2026). I-PREDICT employs an N-of-1 trial structure which enables flexible patient-specific therapy while contributing to an aggregated evidence base (Sicklick et al., 2026). On the other hand, basket trials such as DRUP, pool patients based on a shared molecular biomarker rather than cancer type (Hoes et al., 2022; O’Dwyer et al., 2023). DRUP further refines this approach through an adaptive expansion design, whereby cohorts expand if early signals of clinical benefit are observed, allowing for identification of responsive subgroups to yield stronger evidence for off-label use (Verkerk et al., 2026).
- **Benchmarking safety via adaptive treatment dosing and monitoring:** A central concern in off-target repurposing is uncertainty regarding toxicity, particularly when drugs are used for off-label indications. This risk is illustrated in the DRUP study, where 28.4% of patients experienced grade 3 or higher treatment-related adverse events (Verkerk et al., 2026). Low-evidence regimens introduce uncertainty regarding appropriate starting exposure, combination safety and subsequent dose optimisation. I-PREDICT study demonstrates how such risks can be mitigated by embedding low-evidence hypotheses within prospective, systematically monitored frameworks to improve safety. I-PREDICT achieves this by employing reduced starting doses for low-evidence regimens, careful dose escalation based on individual tolerance – “start low and go slow”, and consideration of drug-drug interactions for rational iterative optimisation of combination therapies (Sicklick et al., 2026). Treatment was adapted in real time, with decisions to continue therapy, modify dose, or cease treatment. This iterative loop is critical for patient safety, distinguishing structured repurposing from static off-label use. Despite 66% of I-PREDICT regimens lacking established safety data, toxicity in the study was manageable, demonstrating that even low-evidence novel therapeutic strategies can be safely explored in this way (Sicklick et al., 2026).
- **Parallel orthogonal validation and emerging optimisation platforms:** A key innovation proposed by Walpole et al. is the integration of parallel orthogonal validation platforms, to support therapeutic decision-making (Walpole et al., 2024). Such orthogonal validation was traditionally completed prior to and hence a barrier for commencing treatment; however, this is often impractical in rapidly progressive disease. Instead we propose that orthogonal validation be used to guide first treatment choice but also conducted in parallel with clinical treatment, functioning as a complementary stream of information to inform ongoing treatment adaptation within the iterative monitoring framework:
  a. Drug specificity: As with on-target matching, response may depend on drug-specific pharmacology, signalling redundancy and tumour context. Orthogonal validation can distinguish which of several off-target candidates is most likely to have anti-tumour efficacy, to refine drug selection before committing patients to low-evidence off-target therapy. For example, Antolin et al. (2020) showed substantial heterogeneity in off-target kinase activity across PARP inhibitors, with favourable candidates prioritised using orthogonal target-engagement assays (Antolin et al., 2020). Pemovska et al. (2015) utilised *ex-vivo* phenotypic screening of patient-derived leukaemia cells prior to treatment commencement to identify that axitinib, a known VEGFR inhibitor, was unexpectedly active against BCR-ABL1 with subsequent clinical response in a patient (Pemovska et al., 2015).
  b. Tumour selectivity and minimising toxicity: Orthogonal validation may also rapidly assess for tumour-selectivity and therapeutic window prior to treatment commencement. Snijder et al. (2017) developed “pharmacoscopy” as a method of *ex-vivo* phenotypic screening that utilises immunophenotyping and single-cell imaging to differentially evaluate drug response on malignant vs. non-malignant cells within a mixed patient tissue sample (Snijder et al., 2017). In their pilot of 17 pharmoscopy-guided patients optimised for tumour cell death over healthy cells, 15 responded and 12 achieved a PFS ratio ≥1.3, showing that integrating this form of functional profiling into clinical treatment selection was technically feasible (Snijder et al., 2017).
  c. Real-time treatment decision-making: Recent trials suggest orthogonal validation can be performed within clinically meaningful timeframes and used to actively refine treatment decisions. Kornauth et al. (2022) and Malani et al. (2022) demonstrate patient-derived *ex vivo* phenotypic screen assay turnarounds of five and four days respectively to inform Tumour Board and treatment decisions, with 30 of 56 Kornauth assay-guided patients achieving a PFS ratio ≥1.3, and a 59% response rate among treated patients in Malani et al. (2022); Kornauth et al. (2022). Emerging computational approaches such as Quadratic Phenotypic Optimisation Platform (QPOP) may further improve efficiency of orthogonal validation. First used in refractory multiple myeloma, QPOP replaces exhaustive combinatorial screening with strategic sampling and mathematical optimisation, so that a limited number of experimentally measured drug responses on *ex vivo* patient samples be used to predict the most promising combinations for further testing (Rashid et al., 2018). QPOP has subsequently been applied to relapsed/refractory lymphoma with median six-day turnaround from biopsy-to-report, and rare soft-tissue sarcomas (Goh et al., 2022; Chan et al., 2025). Together, these studies demonstrate that rapid functional testing, augmented by computational optimisation, can provide a real-time parallel stream of evidence to refine drug selection and treatment adaptation within prospective off-target trials.
- **Trial structure as a key enabler of repurposed drug accessibility:** Access to the repurposed therapy may be limited because of regulatory, reimbursement, and insurer barriers (Lin et al., 2026). Embedding ‘off-target’ repurposing within prospective trials then, serves an additional function in enabling accessibility to off-label therapies with a low evidence base. Additionally, trial structures can be explicitly designed with reimbursement in mind. For example, DRUP incorporates a performance-based reimbursement model within its trial structure, in which access to off-label therapies is initially granted within the trial setting, and continued funding is contingent on demonstrated clinical benefit (Van Waalwijk Van Doorn-Khosrov et al., 2019). This approach mitigates financial risk for healthcare systems while enabling early access for patients.

**FIGURE 1:**
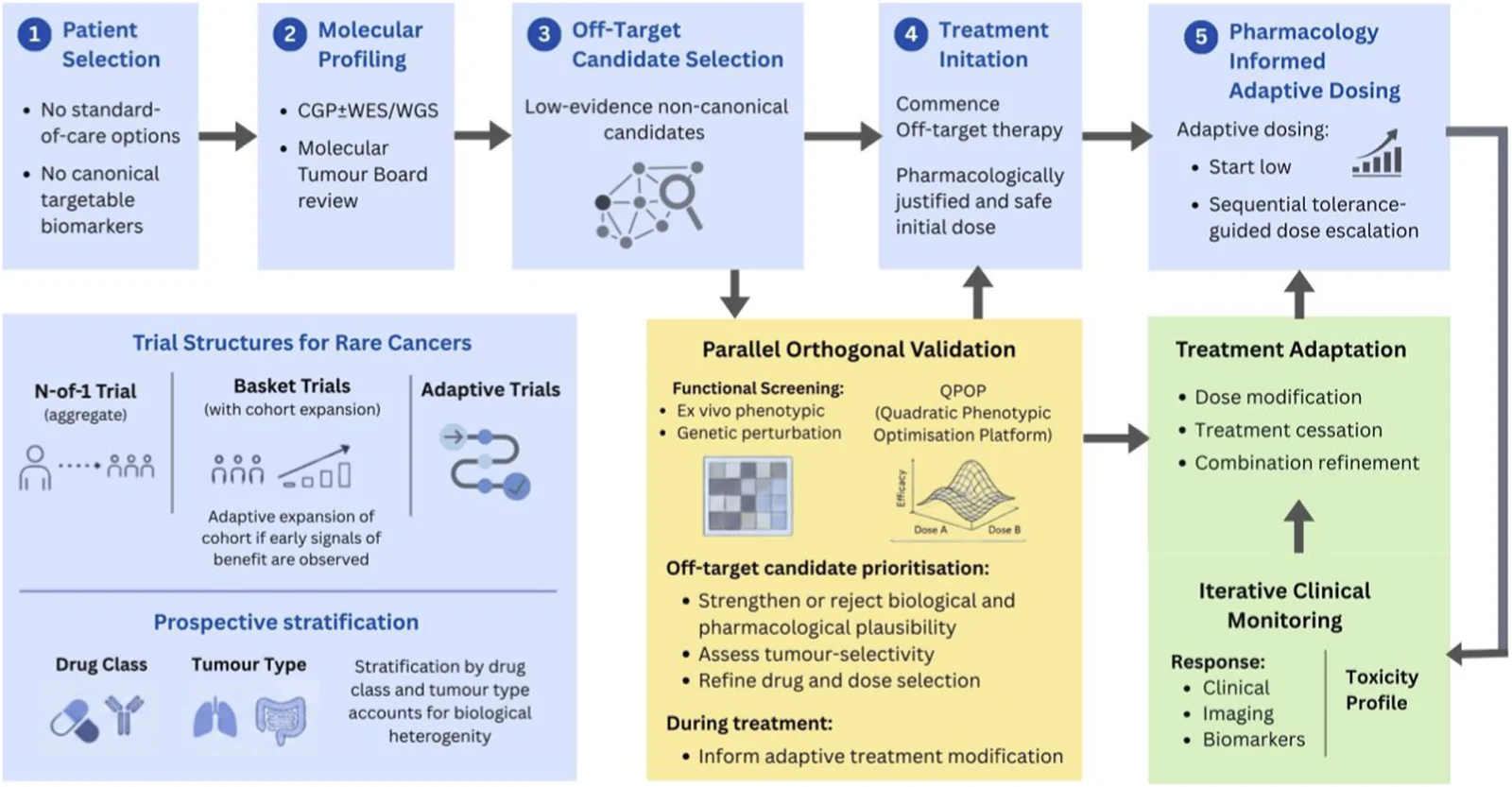
Prospective clinical trial framework for safe implementation of off-target drug repurposing in rare and ultra-rare cancers. Patients lacking standard-of-care treatment options, canonical targetable biomarkers, or access to disease-specific clinical trials are eligible. Low-evidence ‘off-target’ repurposing drug candidates are evaluated within trial structures suited to rare and ultra-rare cancers (N-of-1, basket, and adaptive designs), with prospective stratification by tumour type and drug class to facilitate interpretation of treatment efficacy. Treatment is initiated at a pharmacologically justified and safe starting dose. Parallel orthogonal validation using patient-derived functional approaches such as *ex vivo* phenotypic screening or genetic perturbation studies, and augmented by optimisation platforms such as QPOP, is performed to strengthen or reject biological and pharmacological plausibility, assess tumour selectivity, and refine drug and dose selection. Iterative clinical monitoring is employed to optimise safety, with continuous integration of clinical and validation outcomes to inform adaptive dosing, treatment modification and evidence generation.

## Conclusion

For patients with rare and ultra-rare cancers, conventional therapeutic options are often limited or absent. Despite recent literature questioning the utility of low-evidence genomic matching, off-target repurposing remains a biologically rational strategy to generate therapeutic opportunities when no meaningful alternatives exist. Given the limitations of existing retrospective evidence, rigorous prospective trials are needed to evaluate the efficacy of ‘off-target’ strategies. We contend safe implementation requires embedding low-evidence repurposing candidates within trial designs suited for small heterogeneous cohorts such as basket and N-of-1 designs, which incorporate longitudinal monitoring, and parallel orthogonal validation to strengthen biological and pharmacological plausibility, refine drug and dose selection, and inform adaptive treatment modification over time. By balancing innovation with safety and evidence generation, such frameworks offer a pathway to deliver clinically actionable therapies within meaningful timeframes, while simultaneously advancing the broader evidence base for precision oncology, and mediating equitable access to drugs behind systemic regulatory and financial barriers.

## Data Availability

The original contributions presented in the study are included in the article/supplementary material, further inquiries can be directed to the corresponding author.
